# Robust Small Target Co-Detection from Airborne Infrared Image Sequences

**DOI:** 10.3390/s17102242

**Published:** 2017-09-29

**Authors:** Jingli Gao, Chenglin Wen, Meiqin Liu

**Affiliations:** 1 College of Electrical Engineering, Zhejiang University, Hangzhou 310027, China; gjl991@zju.edu.cn (J.G.); liumeiqin@zju.edu.cn (M.L.); 2 School of Automation, Hangzhou Dianzi University, Hangzhou 310018, China; 3 College of Software Engineering, Pingdingshan University, Pingdingshan 467000, China

**Keywords:** infrared background, target extraction, target refinement, small target co-detection

## Abstract

In this paper, a novel infrared target co-detection model combining the self-correlation features of backgrounds and the commonality features of targets in the spatio-temporal domain is proposed to detect small targets in a sequence of infrared images with complex backgrounds. Firstly, a dense target extraction model based on nonlinear weights is proposed, which can better suppress background of images and enhance small targets than weights of singular values. Secondly, a sparse target extraction model based on entry-wise weighted robust principal component analysis is proposed. The entry-wise weight adaptively incorporates structural prior in terms of local weighted entropy, thus, it can extract real targets accurately and suppress background clutters efficiently. Finally, the commonality of targets in the spatio-temporal domain are used to construct target refinement model for false alarms suppression and target confirmation. Since real targets could appear in both of the dense and sparse reconstruction maps of a single frame, and form trajectories after tracklet association of consecutive frames, the location correlation of the dense and sparse reconstruction maps for a single frame and tracklet association of the location correlation maps for successive frames have strong ability to discriminate between small targets and background clutters. Experimental results demonstrate that the proposed small target co-detection method can not only suppress background clutters effectively, but also detect targets accurately even if with target-like interference.

## 1. Introduction

Infrared small target detection has been widely used in the airborne early warning, infrared guidance, surveillance and tracking and other fields [1,2,3,4]. In these applications, the infrared small targets have the following characteristics: (1) often immersed in strong noises or complex background (cloud clutter, plants and buildings, etc.), (2) with less texture and shape Information, (3) non-cooperative and without fixed law of movement. These characteristics make it very difficult to detect infrared small targets, and it has always been the hot and difficult issue of infrared detection field.

Because of the movement (jitter) of the infrared observation platform or the change of the imaging background, it is difficult to obtain the accurate infrared background by sequential detection methods [5,6,7], because the infrared small targets are easily mistaken for background and vice versa. In this case, the single frame detection methods have received a great attention recently, and are valid for infrared small target detection with static or changing backgrounds [8,9,10]. However, it is difficult to suppress clutters (cloud boundary, targe-like artifacts), which are very similar to real targets from the view of high intensities, because of the limited target information available in a single frame. Fortunately, the commonality of targets in the spatio-temporal domain can be used to build better target detection models and suppress suspected clutters and noise.

To the best of our knowledge, tracklets information are rarely used in existing infrared target detection methods. Note that tracklets information are widely used in tracking problem, in which the target position in the first frame is given in advance [11,12]. However, there is no such prior target information in detection problem in which either a small target exists in a frame or not is still ambiguous.

As discussed above, upon encountering suspected targets or clutters, using the commonality of targets in the spatio-temporal domain is necessary for better detection performance. The commonality features of targets in the spatial domain can be utilized by combining two one-dimensional dense and sparse reconstruction models [13,14]. Different from the one-dimensional dense and sparse reconstruction models [13,14], in this paper we consider the two-dimensional form of dense and sparse reconstruction no longer transforming a matrix to a vector. A two-dimensional dense reconstruction model is proposed based on the global singular value decomposition (SVD) [15], which sets the first few singular values equal to zero and preserves the remaining singular values unchanged. However, this method does not give a general method to select the scope of singular values, and the center-bias mechanism will suppress small targets located at the edges of the image while suppressing clutters or noise. To address this limitation of the global SVD-based reconstruction method [15], we use differences of adjacent singular values to select the proper singular value scope for target extraction, and meanwhile use a sigmoid function to regularize the singular values in order to suppress the background components. The intuition is that each singular value indicates the ability of the corresponding sub-image to approximate the original image. In [8,16], the authors give one-dimensional sparse reconstruction models based on the patch-image model. However, these methods have the following limitations: (1) The detection performance depends largely on the patch size (it was set to 50×50 in [8] or 51×51 in [16]), and the patch vectorization and the pixel reconstruction from overlapped patches could also increase the running time of the algorithm. Moreover, in the patch-image model, one target may appear in different locations of several aligned patches, and after vectorization the intrinsic structure and correlations in the image could be broken, which could influence the separation of target and background later; (2) The algorithm uses L1-norm to measure the sparsity of small targets, but L1-norm treats each pixel independently in terms of intensity, thus the pixels with higher intensities (cloud border, artifacts), are easily mistaken for target pixels, and difficult to be removed through a global threshold [8]. Due to our observation, in an infrared background image, columns (rows) also have non-local self-correlation property and columns (rows) in distant locations are approximately linearly correlated with each other. Hence, to address the first limitation of the patch-image model, we directly consider each column (row) of an image as a column (row) of the observation matrix instead of dividing the image into patches and forming a patch vectorization matrix. Thus, we refer the proposed sparse reconstruction model as a global sparse reconstruction model. Moreover, we exploit entry-wise prior in the sparse reconstruction model to better separate targets from complex backgrounds. The intuition behind the entry-wise prior is that, each pixel in a target should be weighted differently according to its local weighted entropy which measures the local difference between the target and neighboring background. Thus, both the local target features and the global background features are incorporated into the proposed sparse reconstruction model.

For each frame, to increase the confidence level that candidates are real targets, correspondence between suspected targets obtained by dense and sparse reconstructions is conducted to suppress clutters and false alarms further. As we know, the target region in an infrared image has striking discontinuity with the surrounding background. However, due to our observation, the pixels with higher intensities (cloud border, artifacts) as a whole also have this property. Because of the limited target information available in a single frame, these targe-like false alarms could also be detected as real targets. In order to suppress false alarms further, especially the highly suspected targets, in this paper we adopt multiple frame target refinement by tracklets association, based on the facts that real targets and false alarms have different movement characteristics, and false alarms should not be temporally continuous between successive frames like real targets. Due to that the spatio-temporal target commonality is used to refine the rough detection result of each frame in this paper, thus we refer to the propose method as a target co-detection model.

In this paper, we propose a novel infrared target co-detection model that combines the self-correlation features of backgrounds and the commonality features of targets in the spatio-temporal domain to detect infrared small targets in a sequence of images with complex backgrounds. In the first step, the dense reconstruction model is proposed to extract a coarse target map with benefit of regularization of singular values. In the second step, we design a sparse reconstruction model to extract a sparse target map. In the third step, the correspondence between suspected targets of two types of target maps are conducted to suppress clutters and noise. In the fourth step, the tracklets are associated to suppress false alarms and form trajectories which are used to confirm targets for each frame.

The contributions of this paper are summarized as: (1) A dense target extraction method based on regularization of singular values is proposed. Due to the introduction of a sigmoid function, the background components in the target map can be inhibited further. It should be noticed that we do not minimize the nuclear norm but only use the singular value information; (2) A sparse target extraction method based on entry-wise weighted robust principal component analysis is presented. The entry-wise weight uses the structure prior based on the local difference between the target and neighboring background existing in a natural scene from viewpoint of human recognition, which can promote the complex background suppression effect and keep the small target, and (3) we propose a false alarm suppression and target refinement method based on location correlation of the dense and sparse reconstruction maps for a single frame and tracklet association of the location correlation maps for successive frames. Based on the spatio-temporal commonality features of targets, this method can effectively detect small targets and suppress false alarms as much as possible.

The remainder of this paper is organized as follows. Section 2 reviews the related work from the view of processing units in the target detection. Section 3 presents our detection approach comprising of single frame target extraction and multiple frame target refinement. The evaluation on real infrared data set and comparisons are presented in Section 4. Conclusions are given in Section 5.

## 2. Related Work

In recent years, many infrared small target detection methods have been proposed for different applications. According to the processing units in the detection process, we categorize these approaches into pixel-wise, patch-image, and whole-image groups. As discussed later, each group has its own characteristic. The pixel-wise detection methods usually estimate one pixel at a time based on its local neighborhood or its temporal profile, so they could make better use of local difference between the current pixel and its neighborhoods, but are not suitable for the cases when the background scene in a sequence changes fast and weak dim targets are contained in a single image. Besides, the patch-image detection methods calculate each patch in light of the patch set which consists of low-rank and sparse parts, could suffer from more running time caused by vectorization and is also not suitable for detection of weak dim targets with complex background, and the non-local background patches could help separating targets from the patch set. Also according to low-rank matrix approximation, the whole-image detection methods could estimate a whole image from a sequence or a single image, thus the global background feature is used in separating targets from the background, but the whole-image model could also suffer from the problems of vectorization, fast changing background and low signal-to-clutter ratio.

### 2.1. Pixel-Wise Detection Methods

Besides some classical pixel-wise detection methods, such as the topHat method [17], the maxMean and maxMedian methods [18], more pixel-wise approaches have been proposed recently. In [9], the authors proposed an effective small target detection approach according to the contrast mechanism of human vision system and derived kernel model, therein the local contrast measure was defined to compute the dissimilarity between the current location and its neighborhood. In [19], the authors presented local mutation weighted information entropy to suppress background and enhance the gray value of targets. In [10], inspired by the concept of local difference, the authors proposed a weighted local difference measure for the detection of infrared small targets. In addition, in [20], the authors developed a multiscale facet model to enhance targets and then used the multiresolution representation to reduce the false alarm rate. A fast-saliency method based on the facet model was presented for real-time infrared small target detection, and therein the facet kernel operator was designed and used in separating small targets from the background [21]. A biologically inspired method named multiscale patch-based contrast measure was proposed for small infrared target detection, which could increase the contrast between target and background [22]. Furthermore, other pixel-wise detection methods are also proposed using temporal information. Suggested by the singular value decomposition, a temporal filter was developed for dim target detection in evolving cloud clutters [23]. A nonlinear adaptive filter was proposed to detect infrared moving dim targets, and has high performance in removing large fluctuations on temporal profiles that are caused by evolving clutters [5]. By combining spatial and temporal information together, a target detection method was introduced using spatial bilateral filter and temporal cross product, which are respectively used to extract the spatial target information and the features of temporal profiles [6]. Subsequently, a spatial-temporal bilateral filter was presented to detect target trajectories, by extracting spatial and temporal target information simultaneously [7]. As discussed above, pixel-wise detection methods use a local region or a temporal profile to extract target information under the assumption that the target location has conspicuous discontinuity with the nearby background. However, when the the imaging background changes fast or the background has many types of clutters, jamming objects and noises are still the key factor to influence the detection performance.

### 2.2. Patch-Image Detection Methods

In [8], the authors proposed an infrared patch-image (IPI) model for target detection in a single frame. In the IPI model, a frame is divided into small patches and the patches are stacked as columns of a new matrix for robust principal component analysis (RPCA). The intuition behind the IPI model is that the local patches in distant regions in an infrared background image could be approximately linearly correlated with each other. Subsequently, the IPI model inspires much related work [16,24]. In [24], the authors generated an image patch set according to multi-scale transform and patch transform, and every patch was given an individual regulation weight which was computed by combining the information of patch size, patch entropy and target saliency level. In [16], the authors generated an image patch set according to the same scheme in [8], and also stacked all the patches as columns of a matrix, and each patch is given an individual regulation weight based on the steering kernel. However, one target may appear in different locations of several aligned patches, so adding the steering kernel at the central position is not always applicable. As a whole, the patch-image models describe the sparsity of small targets with L1-norm, and the cloud borders or target-like artifacts which have similar intensities with targets are easily mistaken for target pixels. In addition, the performance of IPI models depends on the patch size (it was set to 50×50 in [8] or 51×51 in [16]), and when the patch has a higher dimension, the vectorization in patch-image model needs more computation time.

### 2.3. Whole-Image Detection Methods

In [15], the authors proposed a visible image saliency detection approach based on SVD. The intuition behind this approach is that the large singular values mainly indicate the non-salient background information and slight saliency information, while the intermediate singular values indicate most or even all of the saliency information, and the small singular values contain little or even none of the saliency information. However, this approach does not give a general selection scheme for the scope of singular values which indicates the salient components, and the used center-bias mechanism could suppress small targets at the edges of an infrared image. In [25,26], the authors considered the low-level vision problems where a priori target rank information is available in advance, and minimized the partial sum of singular values instead of minimizing the nuclear norm [27]. In [25,26,27], the individual frames are stacked as columns of a matrix before performing RPCA and each frame is seen as an independent entity. RPCA-based background modeling for static video sequence assumes that these background variations are low-rank and the foreground activity is sparse due to spatially localized. When the background changes fast, it is difficult to obtain accurate backgrounds and then the target regions.

## 3. The Proposed Method

**Figure 1:**
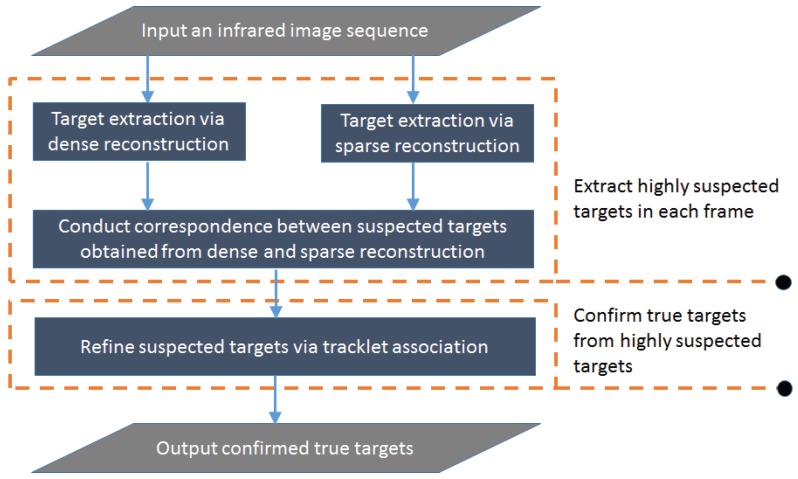
Flow chart of the proposed method.

In this section, we aim to design an infrared small target detection system which consists of two parts as shown in Figure 1. The first part aims to extract highly suspected targets in each frame, and the second part is to confirm true targets from highly suspected targets. The first step of the first part is to suppress complex backgrounds (such as clouds, plants, and strong noises), and detect the suspected targets from a single frame using dense and sparse computation models separately. The second step of the first part is to associate the dense and sparse reconstruction maps obtained by the two computation models in the first step, and suppress false alarms in each frame. Repeating the first step and the second step for consecutive frames, many single-frame detection results can be obtained accordingly. So the second part is to refine the single-frame detection results of different frames based on tracklet association using target location and appearance features.

### 3.1. Infrared Dim Target Model

An infrared image can be usually described as [8]

(1) F(x,y)=B(x,y)+D(x,y)+N(x,y)

where (x,y) denotes the coordinate of a pixel, F(x,y),B(x,y),D(x,y) and N(x,y) are the pixel intensity at coordinate (x,y) for the original infrared image, the background image, the target image, and the random noise image respectively. We can get dense and sparse reconstruction maps from a single image, depending on whether there is a sparse constraint on the target image *D* in the decomposition process.

#### 3.1.1. Frequency Analysis of Infrared Images

It is well known that the singular value decomposition is a powerful tool, and is widely used in latent semantic analysis, recommendation system, defect detection, background suppression and so on [28,29]. The SVD of an infrared image *F* with size m×n can be defined as

(2) $$F=\sum_{i=1}^{r}\sigma_{i}u_{i}v_{i}^{T}$$

where $\left\{u_{i}\right\},\left\{v_{i}\right\}$ and $\left\{\sigma_{i}\right\}$ are the left singular vectors, right singular vectors, and singular values respectively, and $\left\{\sigma_{i}\right\}$ is arranged in descending order.

We can find from (2) that an infrared image *F* can be represented as a sum of different frequency components $\left\{u_{i}v_{i}^{T}\right\}$ regularized by $\left\{\sigma_{i}\right\}$, and the low frequency components correspond to the background part *B* which always changes quite slowly, the medium frequency components correspond to the target part *D* which usually appears as a bright area, and the high frequency components correspond to the noise part *N*. So, each part of an infrared image can be obtained by regularizing proper singular values for different purposes, such as background approximation with low pass filtering, and target detection with band pass filtering.

#### 3.1.2. Low-Rank Analysis of Infrared Images

As discussed above, the infrared background image *B* usually changes slowly and occupies most part of the original image *F*, and the image columns (rows) have the property of non-local self-correlation, i.e., columns (rows) are approximately linearly correlated with each other. Thus the background image *B* can be well approximated by a low-rank matrix.

For a target image, the total number of target pixels is far less than the total number of pixels in the whole image, because of the small size of each target (usually no more than 10×10). So it is reasonable to assume that the target image is sparse.

Based on low-rank property of the background image and sparse property of the target image, the low-rank decomposition model can be used to build up a separation model for small targets and complex backgrounds.

### 3.2. Single Frame Target Extraction

#### 3.2.1. Target Extraction via Dense Reconstruction

A dense reconstruction model is developed here to extract target regions in an infrared image. For an original infrared image *F*, we can construct filters $v_{i}$ (orthogonal bases of row space) by performing SVD on *F*. Based on these filters, we can compute a one-dimensional response $R_{i}^{r}$ by filtering infrared image *F*

(3) $$R_{i}^{v}=\frac{1}{\sigma_{i}}Fv_{i}=u_{i}$$

Similarly, we can obtain a one-dimensional response $R_{i}^{u}$ by filtering infrared image *F* with filters $u_{i}$ (orthogonal bases of column space)

(4) $$R_{i}^{u}=\frac{1}{\sigma_{i}}F^{T}u_{i}=v_{i}$$

Combining Equations (3) and (4), we can compute a two-dimensional response $R_{i}$

(5) $$R_{i}=R_{i}^{v}{\left(R_{i}^{u}\right)}^{T}=u_{i}v_{i}^{T}$$

Equations (3)–(5) show that a pair of filters $\left(u_{i},v_{i}\right)$ can be used to generate a two-dimensional response $R_{i}$ which just corresponds to one frequency component of infrared image *F*. To better utilize multiple frequencies of *F* to extract target information, we choose to combine the obtained two-dimensional responses in a reasonable bound together to obtain the final target map

(6) $$D^{d}=\sum_{i=l}^{h}\rho_{i}R_{i}=\sum_{i=l}^{h}\rho_{i}u_{i}v_{i}^{T}$$

where *l* denotes the low cut subscript, *h* denotes the high cut subscript, and $\rho_{i}$ denotes a linear weight or non-linear weight. When $\rho_{i}$ equals to $\sigma_{i}$, Equation (6) degenerates to Equation (2), up to the low and high cut subscripts. In Equation (6), $R_{i}$ denotes a two-dimensional response of dense filters $v_{i}$ and $u_{i}$, and all its elements is weighted by only one weight $\rho_{i}$, thus the map $D^{d}$ is a global dense representation for infrared targets.

For each response $R_{i}$, the background clutter is still a key factor to influence the final target map, thus the corresponding weight $\rho_{i}$ should be regularized to suppress the background further. In Equation (6), weight $\rho_{i}$ is defined as the logistic sigmoid function of $\sigma_{i}$

(7) $$\rho_{i}=\frac{1}{1+exp(-\sigma_{i})}$$

As mentioned above, each singular value indicates the ability of the corresponding response to approximate the original image. Thus, the singular values can be used to estimate parameters *l* and *h*. Note that the first component always corresponds to the main part of the background, so we do not consider the first singular value in computing *l* and *h*. Let ${\hat{\sigma }}_{i}=\sigma_{i}-\sigma_{i+1},\;i=2,\ldots ,r-1$, and ${\bar{\sigma }}_{1}=\frac{1}{r-1}\left({\hat{\sigma }}_{2}+\cdots +{\hat{\sigma }}_{r-1}\right)$, we can compute *l* using the following equation

(8) $$l=\max_{i}\;{\left\{{\hat{\sigma }}_{i}>\zeta_{1}{\bar{\sigma }}_{1}\right\}}_{i=1}^{r-1}+1$$

A similar consideration can be used to obtain the parameter *h*. Let ${\bar{\sigma }}_{2}=\frac{1}{r-l}\left({\hat{\sigma }}_{l}+\cdots +{\hat{\sigma }}_{r-1}\right)$, we can calculate *h* using the following equation

(9) $$h=\max_{i}\;{\left\{{\hat{\sigma }}_{i}>\zeta_{2}{\bar{\sigma }}_{2}\right\}}_{i=l}^{r-1}$$

$\zeta_{1}$ and $\zeta_{2}$ are scaling factors.

For the final target map $D^{d}$, a small part of background clutter and noise is still required to be removed, because $D^{d}$ comprises of a series of responses $\left\{R_{i}\right\}$ which is regularized by a global weight $\rho_{i}$. In fact, the remaining clutter and noise is not necessarily to be gaussian. Therefore, we use Chebyshev’s theorem to remove the clutter and noise in $D^{d}$, and set Th=μ+cτ as the global threshold, where μ and τ denote the mean and standard deviation of $D^{d}$, and *c* is a positive number and denotes the multiple of standard deviations [30].

#### 3.2.2. Target Extraction via Sparse Reconstruction

As discussed above, it can be concluded from the view of low-rank representation that the task of target map computation can be formulated as a convex optimization problem:

(10) $$\min_{B,D}{∥B∥}_{*}{+\lambda ∥W\circ D∥}_{1},\;s.t.\;{∥F-B-D∥}_{F}^{2}\leq \epsilon$$

where ${∥.∥}_{*}$ denotes the nuclear norm of a matrix, ${∥.∥}_{F}$ is the Frobenius norm of a matrix, ${∥.∥}_{1}$ represents the sum of absolute values of matrix elements, ∘ denotes the entrywise product of the weighting matrix *W* and the target image *D*, λ>0 is a regularization parameter which controls the tradeoff between the background image *B* and the target image *D*, and ϵ>0 is the upper bound of noise energy.

By introducing a multiplier μ>0, the optimization problem (10) can be relaxed as:

(11) $$\min_{B,D}{∥B∥}_{*}{+\lambda ∥W\circ D∥}_{1}+\frac{1}{2\mu }{∥F-B-D∥}_{F}^{2}$$

It can be shown that for some proper value μ(ϵ), any solution of (11) is equivalent to the solution of (10) [27]. To achieve superior convergence, the accelerated proximal gradient (APG) algorithm with a continuation technique on μ is used to solve (11) [31,32,33]. The convex optimization problem (11) can be decomposed into two subproblems that minimize *B* and *D* respectively (for details please see the appendix):

(12) $$B_{k+1}=\arg\min_{B}{\{∥B∥}_{*}+\frac{1}{\mu }∥B-Y_{k}^{B}+\frac{1}{2}\left(Y_{k}^{B}+Y_{k}^{D}-F\right){∥}_{F}^{2}\}$$

(13) $$D_{k+1}=\arg\min_{D}{\{\lambda ∥W\circ D∥}_{1}+\frac{1}{\mu }∥D-Y_{k}^{D}+\frac{1}{2}\left(Y_{k}^{B}+Y_{k}^{D}-F\right){∥}_{F}^{2}\}$$

The subproblems (12) and (13) can be solved by the following equations respectively [34,35]

(14) $$B_{k+1}=B_{\frac{\mu }{2}}\left(Y_{k}^{B}-\frac{1}{2}\left(Y_{k}^{B}+Y_{k}^{D}-F\right)\right)$$

(15) $$D_{k+1}=D_{\frac{\mu \lambda W}{2}}\left(Y_{k}^{D}-\frac{1}{2}\left(Y_{k}^{B}+Y_{k}^{D}-F\right)\right)$$

where $B_{\frac{\mu }{2}}\left(G_{k}^{B}\right)=U\mathrm{diag}(\max$$\left\{\sigma_{i}-\frac{\mu }{2},0\right\})V^{T}$ in which U,V and $\left\{\sigma_{i}\right\}$ are generated by the singular value decomposition of $G_{k}^{B}=Y_{k}^{B}-\frac{1}{2}\left(Y_{k}^{B}+Y_{k}^{D}-F\right)$, and $D_{\frac{\mu \lambda W}{2}}\left(G_{k}^{D}\right)=\mathrm{sign}$$\left(G_{k}^{D}\right)$max(abs$\left(G_{k}^{D}\right)-\frac{\mu \lambda W}{2},0)$ in which $G_{k}^{D}=Y_{k}^{D}-\frac{1}{2}\left(Y_{k}^{B}+Y_{k}^{D}-F\right)$. The details of the solution via APG is described in Algorithm 1.

**Algorithm 1:** Target map extraction by APG. **Input:** Infrared image F,λ,W. **Output:**
$B=B_{k},D^{s}=D_{k}$   1: $B_{0}=B_{-1}=0;D_{0}=D_{-1}=0;t_{0}=t_{-1}=1;\mu_{0}>0;\bar{\mu }>0.$   2: **while** not converged **do**   3:  $Y_{k}^{B}=B_{k}+\frac{t_{k-1}-1}{t_{k}}\left(B_{k}-B_{k-1}\right)$   4:  $Y_{k}^{D}=D_{k}+\frac{t_{k-1}-1}{t_{k}}\left(D_{k}-D_{k-1}\right)$   5:  $B_{k+1}=B_{\frac{\mu }{2}}\left(Y_{k}^{B}-\frac{1}{2}\left(Y_{k}^{B}+Y_{k}^{D}-F\right)\right)$   6:  $D_{k+1}=D_{\frac{\mu \lambda W}{2}}\left(Y_{k}^{D}-\frac{1}{2}\left(Y_{k}^{B}+Y_{k}^{D}-F\right)\right)$   7:  $t_{k+1}=\frac{1}{2}+\frac{1}{2}\sqrt{4t_{k}^{2}+1}$   8:  $\mu_{k+1}=\max\left(0.9\mu_{k},\bar{\mu }\right)$   9:  k=k+1 10: **end while**

The computation of the entry-wise weight matrix *W* is based on the local difference features between the target and neighboring background, and these local difference features could be well measured by the local weighted entropy [19]. For a pixel F(x,y) which has a small neighborhood containing *n* kinds of gray values $f_{1},f_{2},\ldots ,f_{n}$, its local weighted information entropy is expressed as

(16) $$H\left(x,y\right)=-\sum_{i=l}^{n}{\left(f_{i}-F\left(x,y\right)\right)}^{2}p_{i}\log p_{i},p_{i}=\frac{n_{i}}{m\times n}$$

where $n_{i}$ is the number of gray value $f_{i}$ in the neighborhood. Then *H* is convolved with a two-dimensional Gaussian operator Ga(x,y)=exp$\left\{-\frac{1}{2\sigma_{0}^{2}}\left(x^{2}+y^{2}\right)\right\}$ to generate a new version $\bar{H}$. Consequently the weighting matrix W is computed as $\alpha (1-\bar{H})$, where α denotes a regularization parameter which controls the prior impact in the weight matrix.

#### 3.2.3. Target Confirmation via Location Correlation

In order to extract as many highly suspected targets as possible, we use the location cue to remove false alarms and validate candidates in each frame. The intuition is that real targets though obtained by different methods should be located at the same position, but the random noise is not necessarily so. Therefore, through location correlation, some random noise should be suppressed and the ones occurring both in the dense and sparse reconstruction maps may be real targets with high probability.

Suppose that the regions obtained from $D^{d}$ and $D^{s}$ are denoted by $G_{j}^{d}$ with coordinate set $X_{j}^{d}$ and $G_{i}^{s}$ with coordinate set $X_{j}^{s}$ respectively, each region corresponding to a suspected target, and that $D^{c}=0$ has the same size with $D^{d}$ and $D^{s}$. The main steps of target confirmation are described as follows:

1. For each $G_{i}^{s}$ in the target map $D^{s}$, we find $G_{j}^{d}$ in $D^{d}$ whose coordinates are overlapped with that of $G_{i}^{s}$, namely $X_{j}^{d}\cap X_{i}^{s}\neq \emptyset$.
2. For each successfully correlated pair $\left(G_{i}^{s},G_{j}^{d}\right)$, we select pixels in $D^{d}$ with coordinates $X_{i}^{s}$ as the correlation result, namely $D^{c}\left(X_{i}^{s}\right)=D^{d}\left(X_{i}^{s}\right)$.

Note that in step 2, we choose the pixels with coordinates $X_{i}^{s}$ in the dense reconstruction map $D^{d}$. This step could not only avoid the drawback of L1-norm target measure, which treats each pixel independently in terms of intensity and weakens the intensities of the boundary target pixels, but also avoid the drawback of dense reconstruction, which enlarges the target area by combining nearby false alarms together. In essence, the dense reconstruction extracts suspected targets from the view of L2 norm that measures the minimal residual, and thus the boundary target pixels obtained from the dense reconstruction have more bright values than the ones obtained by the sparse reconstruction.

### 3.3. Multiple Frame Target Refinement

After obtaining candidates from each target map $D_{k}^{c}$, we generate suspected target tracklets based on target location and appearance features of consecutive frames, then perform non-maxima suppression to remove false tracks formed by noisy or clutter, finally refine the suspected targets in each target map $D_{k}^{c}$ according to the obtained tracks.

Suppose that the candidates in the target map $D_{p}^{c}$ and $D_{k}^{c}$ are denoted by $G_{j}^{p}=\left\{x_{j}^{p},y_{j}^{p},E_{j}^{p}\right\}$ and $G_{i}^{k}=\left\{x_{i}^{k},y_{i}^{k},E_{i}^{k}\right\}$ respectively. Note that the italic symbol *k* denotes the image index, and the upright symbol p indicates the association result before the *k*th frame. For each candidate, *E* represents the energy of pixels, and (x,y) denotes the average coordinate of pixels. Hence, the tracklets can be generated by repeating linking $\left\{G_{j}^{p}\right\}$ and $\left\{G_{i}^{k}\right\}$ together, whereas $\left\{G_{j}^{p}\right\}$ is updated with each association. The details of generating tracklets are described as follows:

1. We set $D_{p}^{c}=D_{1}^{c},\left\{X^{t}=\left(x_{1}^{t},y_{1}^{t}\right)\right\},\;k=2$.
2. For each $G_{i}^{k}$ in the target map $S_{k}^{c}\left(k\leq L\right)$, we select $\left\{G_{j}^{p}\right\}$ in $D_{p}^{c}$ within a circular gate of $G_{i}^{k}$.
3. For each $G_{i}^{k}$, if $E_{i}^{k}>E_{\theta }$, we keep $G_{i}^{k}$ in $D_{k}^{c}$ and generate $\left\{\left(\left(x_{j}^{p},y_{j}^{p}\right),\left(x_{i}^{k},y_{i}^{k}\right)\right)\right\}$ as small tracklets, here $E_{\theta }$ denotes the average energy of all candidate regions.
4. Update each track $X^{t}$ with a proper tracklet selected from the set $\left\{\left(\left(x_{j}^{p},y_{j}^{p}\right),\left(x_{i}^{k},y_{i}^{k}\right)\right)\right\}$.
5. We set $D_{p}^{c}=D_{k}^{c}$, k=k+1.
6. Repeating the above steps until *k* is greater than *L*, we finally obtain tracks $\left\{X^{t}\right\}$.

After obtaining trajectories through the above process, a non-maximum suppression (NMS) scheme is used to prune false trajectories. For each trajectory $X_{t}$, a displacement gain measure $\Delta_{t}^{1}$ and a length ratio measure $\Delta_{t}^{2}$ are computed as

(17) $$\Delta_{t}^{1}=\sum_{i}|x_{i}^{t}-x_{i-1}^{t}\left|+\right|y_{i}^{t}-y_{i-1}^{t}|$$

(18) $$\Delta_{t}^{2}=\frac{\mathrm{len}(X_{t})}{L}$$

where $\left(x_{i}^{t},y_{i}^{t}\right)$ and $\left(x_{i-1}^{t},y_{i-1}^{t}\right)$ denote elements of track $X_{t}$, and len(·) is a function to solve the length of track $X_{t}$. Next, the trajectories that not only have greater displacement gain than a threshold $\theta_{1}$ but also have longer length gain than a threshold $\theta_{2}$, are selected. The intuition is that real targets and false alarms have different motion features, and the trajectories formed by false alarms are diverse from the true trajectories produced by real targets. Finally, each suspected target in the target map $D_{k}^{c}$ is refined by measuring the distance of its centroid to the valid tracks, and the final detection result $D_{k}^{r}$ is obtained accordingly.

## 4. Experimental Section

### 4.1. Experimental Configuration

#### 4.1.1. Data Sets

In order to fairly evaluate the performance of infrared detection methods, a representative data set consisting of three public infrared sequences with different complex backgrounds is used and the detailed features are listed in Table 1. In Sequence 1, the detection is influenced by strong noise, plants and trees [36]. In Sequence 2, the background changes rapidly due to the movement of imaging platform, and a plane moves from the thick cloud region to the sky [37]. In Sequence 3, the detection barrier is noise and changing wispy clouds [11]. On a whole, the data set contains various situations in airborne infrared target detection. Therefore, using the given data set could fairly show the performance of infrared detection methods.

#### 4.1.2. Evaluation Metrics

The main objective of the proposed method is to effectively suppress the background noise and clutters, and then significantly reduce false alarms to improve detection performance. In this paper, the metrics of signal-to-clutter ratio gain (SCRG), background suppression factor (BSF), precision, recall and F-measure (PRF) are used to evaluate the performance of infrared detection methods. More specifically, the SCRG measures the enhancement of targets relative to the backgrounds before and after detection, and is defined as follows [38,39]:

(19) $$\mathrm{SCRG}=\frac{{\mathrm{SCR}}_{\mathrm{out}}}{{\mathrm{SCR}}_{\mathrm{in}}}$$

where ${\mathrm{SCR}}_{\mathrm{out}}$ and ${\mathrm{SCR}}_{\mathrm{in}}$ are the local SCR values computed from the filtered and original images respectively. Moreover, the BSF evaluates the background inhibition degree, and is defined by [40]:

(20) $$\mathrm{BSF}=\frac{\delta_{\mathrm{in}}}{\delta_{\mathrm{out}}}$$

where $\delta_{\mathrm{in}}$ and $\delta_{\mathrm{out}}$ denote the standard deviations of the original and processed images respectively. In addition, the precision and recall reflect the false alarm rate and miss rate respectively, and the F-measure is the weighted harmonic mean of precision and recall [41].

### 4.2. Overall Performance of the Proposed Method

**Figure 2:**
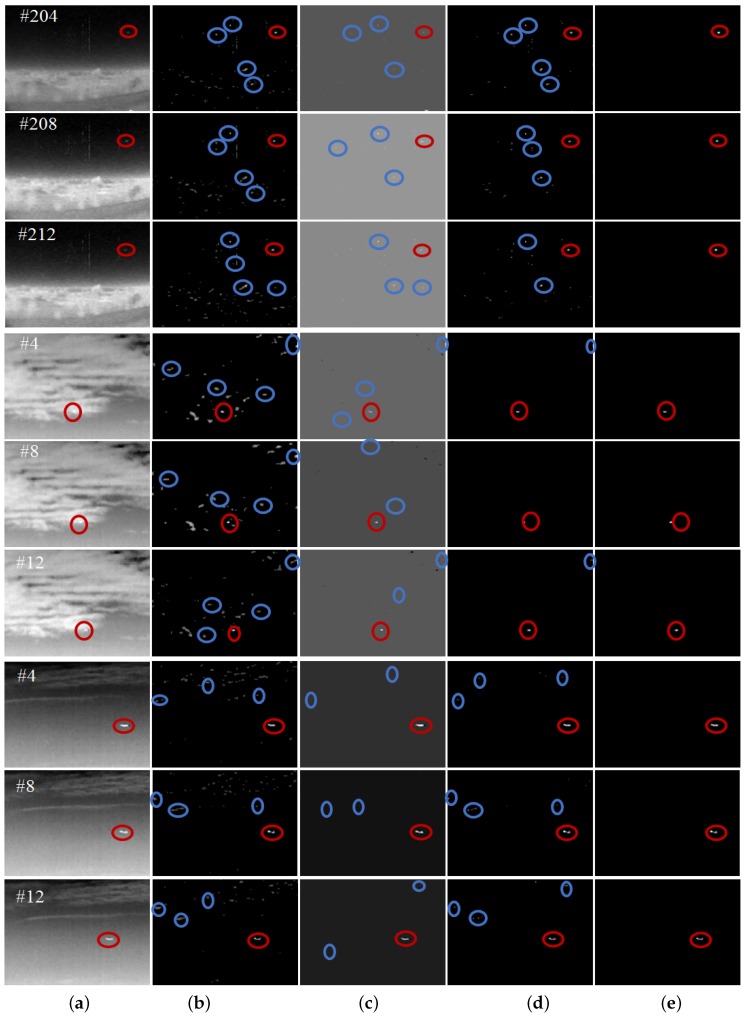
Visual illustration of the procedures of the proposed method. The first three rows, the middle three rows, and the last three rows illustrate the results of the proposed method on Sequence 1, Sequences 2 and Sequence 3 respectively. (**a**) denotes the original images selected from three sequences, (**b**) denotes the dense reconstruction map $D_{k}^{d}$, (**c**) denotes the sparse reconstruction map $D_{k}^{s}$, (**d**) denotes the location correlation map $D_{k}^{c}$ of $D_{k}^{d}$ and $D_{k}^{s}$, and (**e**) denotes the refined map $D_{k}^{r}$.

**Figure 3:**
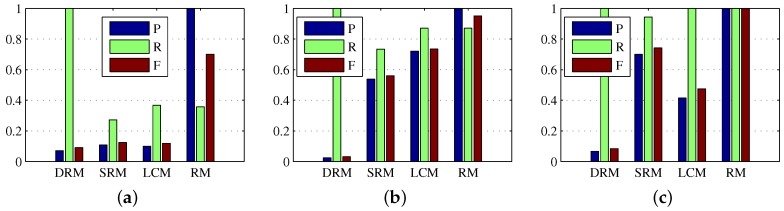
Precision, recall, and F-measure bars for the procedures of the proposed method. (**a**–**c**) denote the results of the procedures of the proposed method on Sequence 1, Sequences 2 and Sequence 3 respectively.

In subsection of dense reconstruction, the scaling factors $\zeta_{1}$ and $\zeta_{2}$ are both set to 1, and the multiple *c* of standard deviations from the mean is set to 3. In subsection of sparse reconstruction, the window of local weighted entropy is set to 5×5, λ is set to 1/sqrt(max(m,n)), $\mu_{0}$ and $\bar{\mu }$ are set to $\sigma_{3}$ and $\frac{1}{r-2}\sum_{i=3}^{r}\sigma_{i}$ respectively, α is set to 2, and $\sigma_{0}$ and the size for the Gaussian filter are set to $\frac{1}{36}\min\left(m,n\right)$ and $\frac{1}{6}\min\left(m,n\right)$ respectively. In addition, in subsection of multiple frame target refinement, the circular gate is set to 15, the displacement gain is set to $\frac{1}{2}\max$$\left(\Delta_{t}^{1}\right)$, and the length threshold is set to 0.6. we first verify the validity of all the procedures of the proposed method, and test the proposed method on all the sequences in the data set with the same configuration parameters. More specifically, the visual illustrations of the dense reconstruction map (DRM) $D_{k}^{d}$, the sparse reconstruction map (SRM) $D_{k}^{s}$, and the location correlation map (LCM) $D_{k}^{c}$ of $D_{k}^{d}$ and $D_{k}^{s}$, and the refined map (RM) $D_{k}^{r}$ are shown in Figure 2, and the quantitative evaluations are given in Table 2 and Figure 3. Note that in Figure 2 the red circles denote the detected real targets, and the blue circles denote some target-like false alarms.

As depicted in Figure 2, $D_{k}^{d}$ and $D_{k}^{s}$ display the ability to reveal the small target in the preliminary results. In addition, the location correlation map $D_{k}^{c}$ of $D_{k}^{d}$ and $D_{k}^{s}$ is shown to contribute to the results further by suppressing the false alarms and enhancing the target. Consistently, as illustrated in Table 2, the SCRG and BSF of $D_{k}^{c}$ was a good tradeoff between that of $D_{k}^{d}$ and $D_{k}^{s}$. Although $D_{k}^{s}$ obtained higher SCRG than $D_{k}^{d}$ did on Sequence 3, it obtained lower SCRG than $D_{k}^{d}$ did on Sequence 1 and Sequence 2, because the very challenging dataset based on an actual application is diverse and characteristic. Hence, a proper tradeoff based on location correlation is necessary for suppressing false alarms in $D_{k}^{d}$ and keeping target pixels in $D_{k}^{s}$. However, after correlation of $D_{k}^{d}$ and $D_{k}^{s}$, there still exist highly suspected targets in the correlation map $D_{k}^{c}$, and some representatives are labeled by blue circles. So in this paper, we use multiple frame target refinement to suppress these false alarms. From the visual and quantitative results in Figure 2 and Figure 3 and Table 2, the refined map $D_{k}^{r}$ produced the best target detection performance.

### 4.3. Parameters Analysis

**Figure 4:**
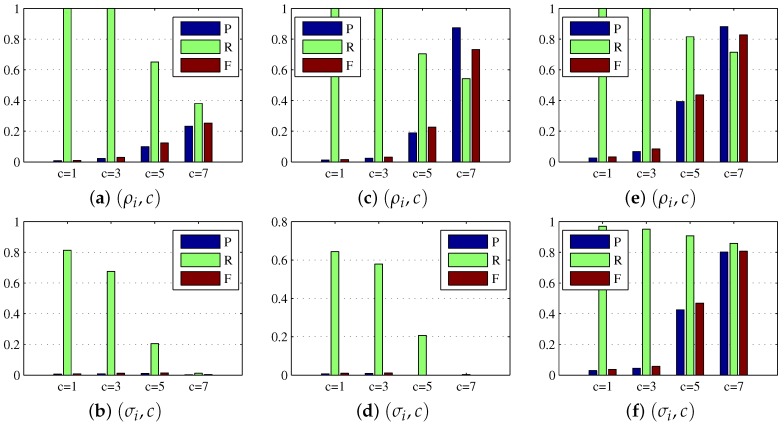
Precision, recall, and F-measure bars for the dense reconstruction maps under different combinations ($\rho_{i},c$) and ($\sigma_{i},c$). (**a**,**b**) denote the results on Sequence 1, (**c**,**d**) denote the results on Sequence 2, and (**e**,**f**) denote the results on Sequence 3.

For the dense reconstruction model, the main relevant parameters include the regularization weight $\rho_{i}$ and multiple *c*. As previously discussed, the regularization weight $\rho_{i}$ helped to eliminate the background influence and enhance the target further, and the multiple parameter *c* controlled the global threshold for target extraction in the dense reconstruction map $D^{d}$. To show the benefits of using $\rho_{i}$ rather than $\sigma_{i}$ in the dense reconstruction model, we consider several combinations $\left(\rho_{i},c\right)$ and $\left(\sigma_{i},c\right)$ in the experiment, and set c=1,3,5,7. As seen from Figure 4a–d and the first four rows of Table 3, for Sequence 1 and Sequence 2, the combinations $\left(\rho_{i},c\right)$ helped target map $D^{d}$ obtain the better precision, recall, and F-measure, and achieve the higher average SCRG and BSF values. Note that the symbol *∞* in Table 3 indicates that the target map obtained by the combination $\left(\sigma_{i},c=7\right)$ is a zero matrix which means that there is no target or background information in the target map, hence the choice of $\rho_{i}$ is superior to $\sigma_{i}$ in the process of target reconstruction. In addition, we found from Figure 4e–f and the last two rows of Table 3 that there was no diverse difference between the results obtained by using combinations $\left(\rho_{i},c\right)$ and $\left(\sigma_{i},c\right)$ on Sequence 3, and the combination $\left(\rho_{i},c\right)$ obtained a slightly worse result than $\left(\sigma_{i},c\right)$. The reason is that $\rho_{i}$ could also suppress the target when suppressing the background, and the background in Sequence 3 is not as complex as that in Sequence 1 and Sequence 2. As a whole, the regularization weight $\rho_{i}$ is more suitable for more complex background suppression than $\sigma_{i}$. As mentioned, *c* is also an important parameter. As shown in Table 3, the BSF increased with increasing *c* value, while the SCRG decreased with increasing *c* value. Hence, an intermediate *c* value could be a good choice. The consistent conclusion can be seen from Figure 4, because for example the case c=7 could cause high miss rate while the case c=1 could result in lots of false alarms.

**Figure 5:**
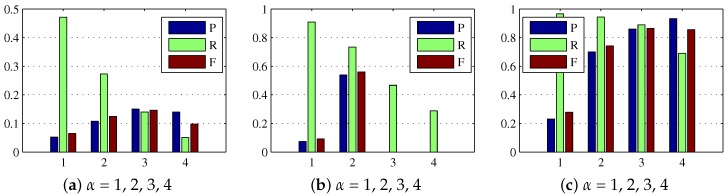
Precision, recall, and F-measure bars for the sparse reconstruction maps under different values α. (**a**–**c**) denote the results on Sequence 1, Sequences 2 and Sequence 3 respectively.

For the sparse reconstruction model, the regularization parameter α controlled the prior impact in the weight matrix. We varied α from 1 to 4 in the experiment, and illustrated the precision, recall, and F-measure in Figure 5. From the illustration, it could be observed that an intermediate α would give a better tradeoff between miss rate and false alarms. For example, the precision, recall, and F-measure of α=4 showed a high miss rate on Sequence 1 and Sequence 2, because the real targets were suppressed by the overlarge prior weight. In contrast, when the low miss rate was guaranteed, the false alarm rate of α=1 is higher than other settings, suggesting that a too small α is also not a proper choice.

**Figure 6:**
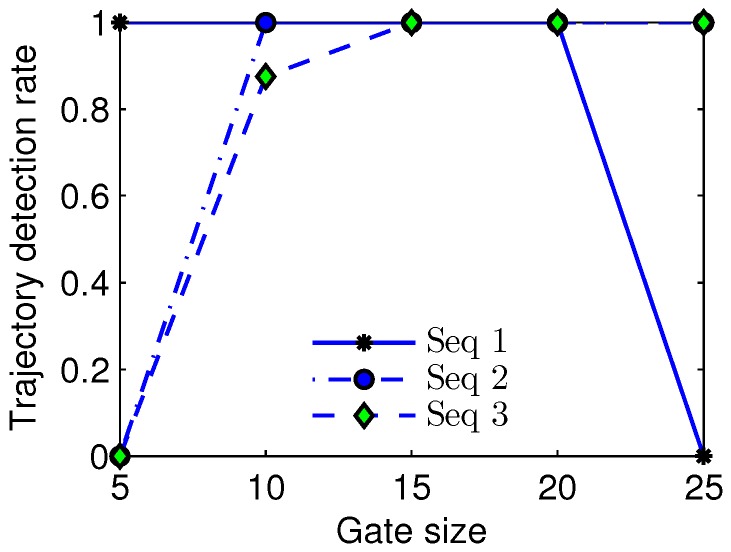
Relationship curve of the trajectory detection rate and gate size.

As described in subsection of multiple frame target refinement, a trajectory comprises of small tracklets which mainly depend on the gate size δ of $G_{i}^{k}$. We varied the gate size δ from 5 to 25 in the experiment, and showed the relationship between the trajectory detection rate and gate size in Figure 6. It could be observed that a small gate size δ<10 could result in a low trajectory detection rate on Sequence 2, a small gate size δ<15 could cause a low trajectory detection rate on Sequence 3, and a large gate size δ>20 could lead to a low trajectory detection rate on Sequence 1, because the real targets in Sequence 1, Sequence 2, and Sequence 3 have different velocities which can be inferred from Figure 2. For example, because the target in Sequence 1 has the lowest velocity, a very large gate size could lead to false association between the current frame and the former frame, and moreover the target in Sequence 3 has the highest velocity, thus a very small gate size could result in association failure between consecutive frames. From the illustration, we can find that the interval [15,20] could be a proper scope of the gate size for Sequence 1, Sequence 2, and Sequence 3.

### 4.4. Comparison with State-Of-The-Art Approaches

**Figure 7:**
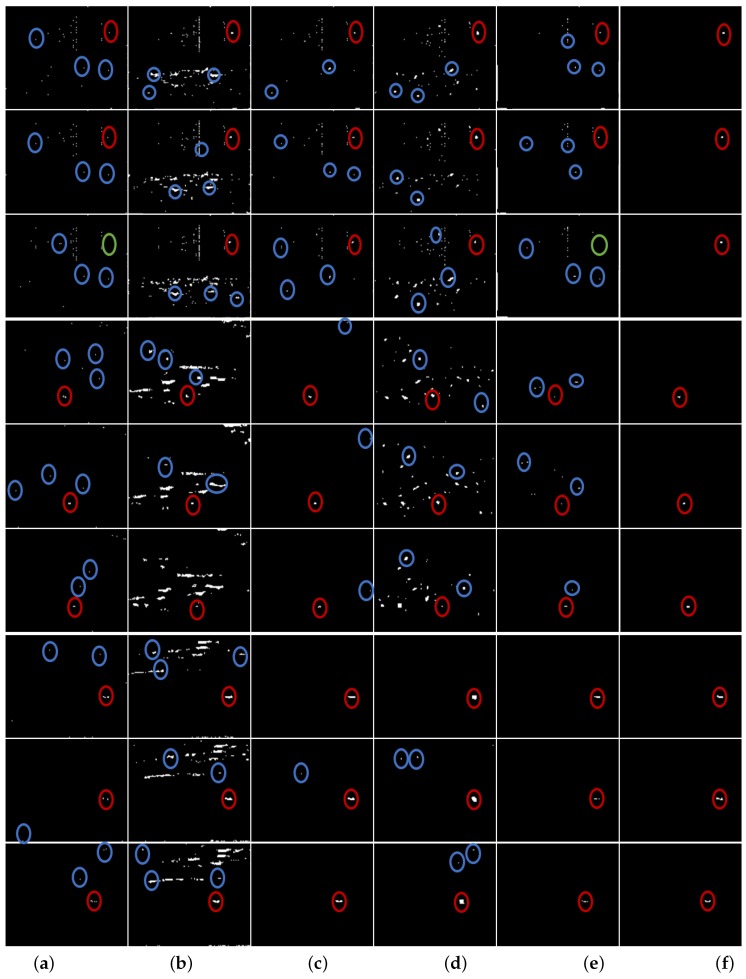
Visual illustration of the results of the proposed method and other existing methods. The first three rows, the middle three rows, and the last three rows illustrate the results of the proposed method and other existing methods on Sequence 1, Sequences 2 and Sequence 3 respectively. (**a**) denotes the detection results of MM method, (**b**) denotes the detection results of TH method, (**c**) denotes the detection results of IPI model, (**d**) denotes the detection results of MPC method, (**e**) denotes the detection results of WLD method, and (**f**) denotes the detection results of the proposed method.

**Figure 8:**
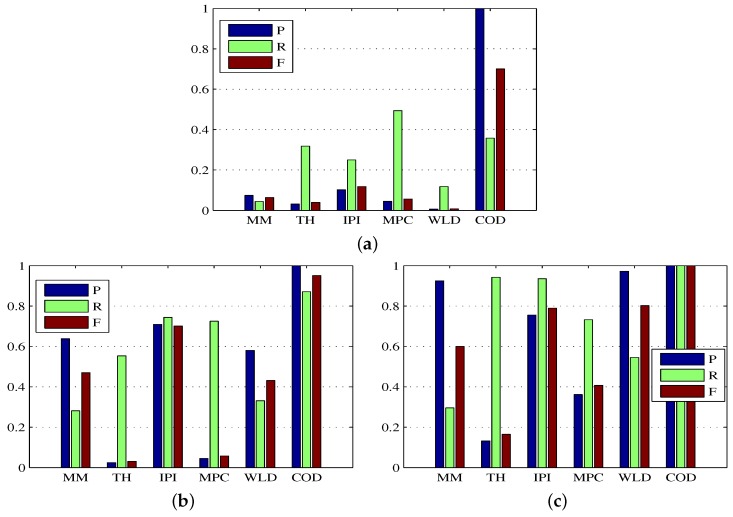
Precision, recall, and F-measure bars for the proposed method and other existing methods. (**a**–**c**) denote the results of the proposed method and other existing methods on Sequence 1, Sequences 2 and Sequence 3 respectively.

In order to show the performance of the proposed method, we selected five state-of-the-art infrared small target detection methods, including two classical methods (MM [18], TH [42]), and three recent methods (IPI [8], MPC [22], WLD [10]) based on the patch-image model, the multiscale contrast measure and the local difference measure. As a whole, the adopted comparison approaches are representatives of the advanced level of infrared small target detection at present. The detailed parameter settings used in the experiments are described in Table 4 for reproduction. The detection results of the proposed method and the existing methods are visually shown in Figure 7 in which the red circles denote the detected real targets, the blue circles denote the false alarms, and the green circles indicate that the real targets were lost. Note that the results of the existing methods in Figure 7 are obtained using a global threshold μ+3τ, and the original images corresponding to Figure 7 are the same as those of Figure 2. In addition, the quantitative evaluation results are provided in Table 5 and Figure 8.

As depicted in Table 5, the MM achieved higher BSF than TH on Sequence 1, Sequence 2 and Sequence 3, but gave the worst performance in terms of the SCRG and the visual illustration of Figure 7. In addition, the TH and MPC achieved the top two SCRG on Sequence 3, but gave the poor detection results as seen from the visual result of Figure 7 that many target pixels were lost in the detection result of MPC, and that much background cloud was left in the detection of TH. Moreover, the IPI model exhibited excellent background suppression performance, but the SCRG is very low when the background was complex in Sequence 1. Furthermore, the WLD achieved the highest BSF on Sequence 3, but obtained less SCRG which was slightly better than that of MM. As shown in Figure 7 and Figure 8b–c, for the IPI and WLD methods, the false alarms can be well suppressed with an appropriate threshold on Sequence 2 and Sequence 3. However, as shown in Figure 7 and Figure 8a, the existing five state-of-the-art methods still performed poorly on Sequence 1, because a very challenging test sequence based on an actual application (existence of targe-like false alarms as shown in Figure 7) was used for the comparative testing in this paper. Thus, the success of the existing five methods based on only a single image was restricted to its own specific application. Therefore, exploitation of the motion and appearance cues from the image sequences was necessary to further improve the detection performance of a single frame. Although the proposed method do not have the highest SCRG and BSF, based on the visual comparison in Figure 7 and the quantitative comparison in Figure 8, it is clear that the proposed method consistently performed well on all three sequences and outperformed other test methods from the view that all false alarms including the target-like ones in each image are well suppressed.

### 4.5. Computational Complexity

The computational complexity and time for the proposed method and other existing methods were given in Table 6. All the experiments were carried out on a computer with a 3.2 GHz Intel CPU and 4-GB memory. The image size is m×n, the patch-image size in the IPI model is $\bar{m}\times \bar{n}$, β denotes the iteration number of the algorithm, and *p* denotes the pixel number in the support region. As depicted in Table 6, the six test methods differed greatly in running time, though there existed a little difference in the computational complexity. For the MM and TH methods, the time difference is mainly caused by the max operation in MM. For the MPC and WLD methods, the time difference lies in the sort operation in the computation of local entropy. In addition, the IPI method took the longest time in the test methods, the cost is mainly caused by the vectorization and median operations. However, the running time of the proposed method is only about one-fiftieth the time of IPI method. The essential reason is that the non-patch scheme and local prior weight contribute to improving convergence speed. Although the proposed method took more time than the MM, TH and MPC methods did, it is acceptable from the view of detection performance.

## 5. Conclusions

In this paper, a novel infrared target co-detection model, which combines the self-correlation features of backgrounds and the commonality features of targets in the spatio-temporal domain, is proposed to detect small targets in a sequence of images with complex backgrounds. On one hand, the nonlinear weights has been constructed based on the logistic sigmoid function, and has more advantages than weights of singular values in suppressing background and keeping small targets. On the other hand, the entry-wise weight has been designed based on the local weighted entropy, and can extract real targets accurately and suppress background clutters efficiently. Finally, the location correlation of the dense and sparse reconstruction maps for a single frame and tracklet association of the location correlation maps for successive frames are performed to suppress false alarms and confirm suspected targets. The experiments have testified the effectiveness of the proposed co-detection model.

## Figures and Tables

**Table 1:** Details of the evaluation data set.

Sequences	Number	Size	Target Type	Target Details	Background Details
Sequence 1	600	320×240	Helicopter	Low SCR value	Heavy noise backgrounds
				A smaller size	Skyline and plants backgrounds
Sequence 2	30	256×200	Airplane	A long imaging distance	Heavy clouds backgrounds
				Keeping curved movement	Uniform backgrounds
				A changing size with a big range	
Sequence 3	40	256×200	Airplane	A long imaging distance	Wispy cloud backgrounds
				Keeping zigzag-shaped movement	Uniform backgrounds

**Table 2:** Average scores of the procedures of the proposed method.

Sequences	Metrics	DRM	SRM	LCM	RM
Sequence 1	SCRG	2.37	1.71	2.27	2.31
	BSF	7.01	25.44	10.91	19.12
Sequence 2	SCRG	3.25	3.14	3.60	3.60
	BSF	1.89	9.20	6.04	6.51
Sequence 3	SCRG	1.33	1.38	1.34	1.34
	BSF	4.19	8.37	6.67	7.84

**Table 3:** Average scores of the dense reconstruction maps with different combinations.

Sequences	Metrics	$\rho_{i}$$(\sigma_{i}),c=1$	$\rho_{i}\left(\sigma_{i}\right),c=3$	$\rho_{i}\left(\sigma_{i}\right),c=5$	$\rho_{i}\left(\sigma_{i}\right),c=7$
Sequence 1	SCRG	2.86(2.72)	2.60(1.99)	2.27(0.97)	1.77(0.08)
	BSF	4.49(2.56)	7.01(3.42)	12.05(5.08)	18.44(8.81)
Sequence 2	SCRG	2.83(1.79)	3.25(1.60)	3.20(1.12)	2.74(0.03)
	BSF	1.17(0.50)	1.89(0.77)	4.14(*∞*)	6.83(*∞*)
Sequence 3	SCRG	1.37(1.49)	1.33(1.43)	1.25(1.39)	1.18(1.34)
	BSF	2.86(3.87)	4.19(4.71)	6.50(6.24)	7.90(6.81)

**Table 4:** Detailed parameter settings of the six test methods.

No.	Methods	Acronyms	Parameter Settings
1	Max-Mean filter	MM	Support size: 5×5
2	Top-Hat approach	TH	Structure element: disk, radius: 6
3	Infrared patch image model	IPI	Patch size: 50×50, sliding step: 10, $\lambda =1/\sqrt{\max(m,n)}$
4	Multiscale patch-based contrast measure	MPC	N = 2, 3, 4, 5
5	Weighted local difference measure	WLD	L = 4, entropy neighborhood 5×5
6	Proposed co-detection method	COD	$\lambda =1/\sqrt{\max(m,n)}$, entropy neighborhood 5×5

**Table 5:** Average scores of the proposed method and other existing methods.

Sequences	Metrics	MM	TH	IPI	MPC	WLD	COD
Sequence 1	SCRG	0.80	1.99	1.55	2.45	2.27	2.31
	BSF	9.59	5.26	23.34	16.14	9.81	19.12
Sequence 2	SCRG	2.10	2.67	3.13	2.91	2.35	3.60
	BSF	2.30	0.77	9.39	7.44	8.36	6.51
Sequence 3	SCRG	0.78	1.51	1.37	1.45	1.01	1.34
	BSF	6.08	3.54	8.35	7.54	10.37	7.84

**Table 6:** Algorithm complexity and running time of the proposed method and other existing methods.

	MM	TH	IPI	MPC	WLD	COD
Complexity	O(4*mn*)	O(2*pmn*)	O(β*k*$\bar{mn}$log($\bar{mn}$) + *mnp*)	O((*p* + 1)L*mn*)	O((L + *p*)*mn*)	O(*mn*(*p + k* + β*k*log(*mn*)))
Time (s)	8.30	0.17	503.65	0.28	18.20	9.2

## Appendix A

With $X={\left(B,D\right)}^{T},g\left(X\right)={∥B∥}_{*}+\lambda {∥W\circ D∥}_{1}$, and $f\left(X\right)=\frac{1}{2\mu }{∥F-B-D∥}_{F}^{2}$ in (11), thus $\nabla_{B}f\left(X\right)=\nabla_{D}f\left(X\right)=\frac{1}{\mu }\left(B+D-F\right)$, and $\nabla f\left(X\right)={\left(\nabla_{B}f\left(X\right),\nabla_{D}f\left(X\right)\right)}^{T}$, we can obtain

(A1) $$\begin{array}{cc} ∥\nabla f\left(X_{1}\right)-\nabla f\left(X_{2}\right){∥}_{F}= & \frac{1}{\mu }{∥\left[\begin{array}{c} B_{1}+D_{1}-F\\ B_{1}+D_{1}-F \end{array}\right]-\left[\begin{array}{c} B_{2}+D_{2}-F\\ B_{2}+D_{2}-F \end{array}\right]∥}_{F}=\frac{1}{\mu }{∥\left[\begin{array}{c} B_{1}-B_{2}\\ D_{1}-D_{2} \end{array}\right]+\left[\begin{array}{c} D_{1}-D_{2}\\ B_{1}-B_{2} \end{array}\right]∥}_{F}\\ \leq & \frac{1}{\mu }{∥\left[\begin{array}{c} B_{1}-B_{2}\\ D_{1}-D_{2} \end{array}\right]∥}_{F}+\frac{1}{\mu }{∥\left[\begin{array}{c} D_{1}-D_{2}\\ B_{1}-B_{2} \end{array}\right]∥}_{F}=\frac{2}{\mu }{∥X_{1}-X_{2}∥}_{F} \end{array}$$

So *f* is Lipschitz continuous, and the Lipschitz constant $L_{f}=\frac{2}{\mu }$. According to the proximal gradient approach, we could approximate f(X) locally as a quadratic function

(A2) $$\begin{array}{cc} (B_{k+1},D_{k+1})= & X_{k+1}=\arg\min_{X}\{g\left(X\right)+f\left(Y_{k}\right)+<\nabla f\left(Y_{k}\right),X-Y_{k}>+\frac{L_{f}}{2}∥X-Y_{k}{∥}_{F}^{2}\}\\ = & \arg\min_{X}\left\{g\left(X\right)+\frac{1}{\mu }∥X-Y_{k}+\frac{\mu }{2}\nabla f\left(Y_{k}\right)\right){∥}_{F}^{2}\}\\ = & \arg\min_{B,D}{\{∥B∥}_{*}{+\lambda ∥W\circ T∥}_{1}+\frac{1}{\mu }{∥\left[\begin{array}{c} B\\ D \end{array}\right]-\left[\begin{array}{c} Y_{k}^{B}-\frac{\mu }{2}\nabla_{B}f\left(Y_{k}\right)\\ Y_{k}^{D}-\frac{\mu }{2}\nabla_{D}f\left(Y_{k}\right) \end{array}\right]∥}_{F}^{2}\}\\ = & \arg\min_{B,D}{\{∥B∥}_{*}+\frac{1}{\mu }∥B-Y_{k}^{B}+\frac{\mu }{2}\nabla_{B}f\left(Y_{k}\right){)∥}_{F}^{2}+\lambda {∥W\circ D∥}_{1}\\ +\frac{1}{\mu }∥D-Y_{k}^{D}+\frac{\mu }{2}\nabla_{D}f\left(Y_{k}\right){)∥}_{F}^{2}\}, \end{array}$$

and then solve (A2) to update the solution *X*. It can conclude that the problem (A2) is separable and can be decomposed into two subproblems (12) and (13).
